# Physiology-informed use of *Cupriavidus necator* in biomanufacturing: a review of advances and challenges

**DOI:** 10.1186/s12934-025-02643-x

**Published:** 2025-01-22

**Authors:** Michael Weldon, Christian Euler

**Affiliations:** Department of Chemical Engineering, University of Waterloo, Waterloo, Canada

**Keywords:** *Cupriavidus necator* H16, Metabolic engineering, Synthetic biology, Biomanu-facturing, Carbon fixation

## Abstract

Biomanufacturing offers a potentially sustainable alternative to deriving chemicals from fossil fuels. However, traditional biomanufacturing, which uses sugars as feedstocks, competes with food production and yields unfavourable land use changes, so more sustainable options are necessary. *Cupriavidus necator* is a chemolithoautotrophic bacterium capable of consuming carbon dioxide and hydrogen as sole carbon and energy sources, or formate as the source of both. This autotrophic metabolism potentially makes chemical production using *C. necator* sustainable and attractive for biomanufacturing. Additionally, *C. necator* natively fixes carbon in the form of poly-3-hydroxybutyrate, which can be processed to make biodegradable plastic. Recent progress in development of modelling and synthetic biology tools have made *C. necator* much more usable as a biomanufacturing chassis. However, these tools and applications are often limited by a lack of consideration for the unique physiology and metabolic features of *C. necator*. As such, further work is required to better understand the intricate mechanisms that allow it to prioritise generalization over specialization. In this review, progress toward physiology-informed engineering of *C. necator* across several dimensions is critically discussed, and recommendations for moving toward a physiological approach are presented. Arguments for metabolic specialization, more focus on autotrophic fermentation, *C. necator*-specific synthetic biology tools, and modelling that goes beyond constraints are presented based on analysis of existing literature.

## Background

The chemical industry plays a crucial role in society by providing important chemicals to consumers and other industries. It represents 30% of the industrial energy demand worldwide [1] and relies on non-renewable resources that are predicted to run out before the end of the century [2, 3]. While the energy requirements to power chemical processes are an important source of emissions, many of the compounds produced by the chemical industry are also derived from petroleum [4, 5]. The primary petrochemicals—ethylene, propylene, butadiene, benzene, toluene, and *p*-xylene—have embedded carbon that is released and the end of the product lifetime making up between 65 and 73% of their carbon footprint [6]. Therefore, unless the use of alternative feedstocks can meet or exceed fossil fuel-based production in terms of yield, productivity, sustainability, and economic feasibility there will be little incentive to transition away from fossil fuels. Biomanufacturing, in which organisms are used to make valuable products, is emerging as a way to overcome this dependence. Instead of consuming fossil fuels, microbes can be grown in fermentation processes using a variety of feedstocks and can produce a wide range of metabolites, including valuable chemicals. While the variety of naturally available microbes offers diverse options for consuming and producing different compounds, metabolic engineering can be used to control the reactions that take place in the cell to allow greater diversity of substrates and products, and improved production, which is crucial to meeting industrial demands.

Metabolic engineering strategies toward these goals include engineering transcription and translation, notably by modifying origins of replication, promoters, terminators, ribosome binding sites (RBS), and DNA and RNA regulators [7, 8]. Another strategy is compartmentalization, which can utilize fusion proteins, scaffolds, and organelles or cellular compartments. Cofactor availability can also be modulated, as well as the preference of enzymes for specific cofactors. Similarly molecular transport can be controlled by changing the expression of transporters or by changing their substrate preference [8].

Despite the large and growing number of metabolic engineering tools and strategies, the natural capabilities of organisms can still be more useful than and outperform engineered ones, as different organisms are adapted to different niches, which makes them more suitable for certain biotechnology tasks [9]. For use at the industrial scale, these properties should be combined with fast and efficient growth, high product yields, and/or simple and cost-effective fermentation [10]. Previously, non-traditional organisms were difficult to engineer beyond their native capabilities, but the expanding suite of synthetic biology tools for a widening range of organisms opens the door to using the natural properties of these other microbes more rationally [9]. As such, the choice of chassis is more important than ever, and non-traditional organisms must be considered more readily. One of these organisms is *Cupriavidus necator* H16, a Gram-negative chemolithoautotrophic β-proteobacterium. *C. necator* is a soil and freshwater-adapted microbe discovered 60 years ago [11, 12]. It is known as a knallgas bacterium, due to its aerobic hydrogen oxidation ability [11], and it is notably attractive to metabolic engineers for its metabolic adaptability.

This review explores the ways that this feature can and is being utilised while highlighting areas that require refocusing with the physiology of *C. necator* in mind. The research exploring the use of *C. necator* in biomanufacturing is progressing quickly, and its applications are diverse. Therefore, this review will provide an update on the state of the field to summarize the engineering tools and strategies. It will also suggest approaches that should be prioritized to maximise the benefits of using a *C. necator* biomanufacturing chassis by taking advantage of its unique properties. These suggestions will focus on changing the niche of *C. necator* to that of a high-producing industrial strain, on engineering autotrophic metabolism, and on designing robust tools.

## Main text

### Shifting from generalist to specialist

*C. necator* is a facultative chemolithoautotroph, which means it can grow heterotrophically using a variety of substrates, or autotrophically using CO_2_ as its carbon source through the Calvin-Benson-Basham (CBB) cycle [13]. The sugars that *C. necator* can catabolize are fructose and N-acetylglucosamine, which can be converted to fructose [14]. The Entner-Doudoroff (ED) pathway is used to catabolise fructose and gluconic acid. It can also use levulinic acid by converting it to propionyl-CoA and acetyl-CoA [13]. In addition, *C. necator* can grow using acetate, succinate, and a number of aromatic compounds including benzoate, m-hydroxybenzoate, p-hydroxybenzoate, phenol, p-cresol, and L-tryptophan [15]. While this list of potential substrates is not exhaustive, it demonstrates the versatility of *C. necator* carbon metabolism and thus its potential use in a wide range of fermentation conditions and for production of diverse compounds. Despite this heterotrophic growth versatility, autotrophy via CO_2_ consumption is one of the most attractive *C. necator* features for biotechnology, as CO_2_ is a cheap and widely available carbon source, and it is the only truly sustainable carbon source on Earth, whether used directly or via CO_2_-derived biomass.

In the CBB cycle, CO_2_ is fixed through the activity of the inefficient ribulose bisphosphate carboxylase (RuBisCo). To help promote the activity of RuBisCo, cyanobateria have developed a carbon concentrating mechanism (CCM) that accumulates inorganic carbon in the cell, mainly as bicarbonate (HCO_3_^−^). The HCO_3_^−^ is then converted to CO_2_ by a carbonic anhydrase (CA) to concentrate CO_2_ near RuBisCo [16]. While *C. necator* does not have a typical CCM, it does have four CA-like enzymes that help drive CO_2_ fixation by RuBisCo [17]. These four CAs have different but connected roles that are still being elucidated [18]. In *C. necator*, these CA-like enzymes are responsible for concentrating CO_2_, but more importantly, they convert CO_2_ to bicarbonate to meet the needs of autotrophic anaplerotic metabolism [19, 20].

During autotrophic growth on CO_2_, *C. necator* uses hydrogen as its energy source through the action of hydrogenases. Hydrogenases are metalloenzymes—of which there are three classes: NiFe, FeFe, and Fe—that catalyze the oxidation of a hydrogen molecule (H_2_) into two protons (H^+^) and two electrons [21]. *C. necator* expresses four different types of hydrogenases: a soluble hydrogenase (SH), a membrane-bound hydrogenase (MBH), a regulatory hydrogenase (RH), and an actinobacterial hydrogenase (AH) [17]. The SH has attracted the most attention because it is uniquely oxygen-tolerant. It is a tetrameric enzyme that includes a hydrogenase subunit that binds and acts on H_2_, and a subunit that binds NAD + and reduces it to NADH [21].

The other hydrogenases are also of interest because they are, to a lesser extent, also oxygen- tolerant [22–24]. It has been shown that the MBH achieves oxygen tolerance by converting O_2_ and H_2_ to water and releasing two electrons [22]. The oxidation of H_2_ by the MBH is then used to reduce ubiquinone and store the electrons [17]. The RH senses H_2_, transfers the electrons to an unidentified redox cofactor, and then the signal to control the expression of the other hydrogenases is transduced using a histidine protein kinase [17, 25]. The AH is less studied, but it acts more slowly than the others and seems to be active under nutrient-limited conditions [24, 26, 27]. The oxygen-tolerance of these four hydrogenases, which makes *C. necator* a knallgas bacterium, is crucial to the use of this organism as a chassis because it does not require anaerobic conditions to obtain electrons from H_2_ and store them in NADH. Therefore, certain waste streams like flue gas and syngas, which contain O_2_, could be fed directly to *C. necator* to recycle these waste products [28]. In addition, understanding the role of hydrogenases in cofactor balancing is likely critical to successfully engineering redox metabolism in *C. necator*.

*C. necator* can also use formate as a source of carbon and energy. It does so via four metal-dependent formate dehydrogenases (FDH) [29, 30]. One of these enzymes is cytosolic, while the other three are bound on the periplasmic side of the membrane [30]. The soluble FDH oxidizes formate to CO_2_ and reduces NAD + to NADH [31]. It is also O_2_-tolerant like the hydrogenases of *C. necator* [29], so it is similarly relevant to aerobic fermentation. The ability to grow on formate is potentially of great use to biotechnology because formate can be produced electrocatalytically from CO_2_ [32], which may make it a sustainable feedstock. It may help to overcome the inherent mass transfer limitations of gaseous feedstocks like H_2_, which has a relatively low water solubility [33, 34] because formate can be fed as liquid formic acid or as soluble sodium formate. In addition, it provides the cells with both carbon and electrons together rather than having to provide both CO_2_ and H_2_, for example. While this may offer convenience, the fixed ratio of carbon and electrons may limit metabolic flux compared to CO_2_/H_2_ mixtures that could be tuned to drive CO_2_ consumption via an excess supply of electrons. It is important to note that the FDH also catalyzes the reverse reaction oxidizing formate to CO_2_ [29]. This is potentially of importance when engineering carbon flux towards desired products, as it may constrain the amount of flux that can be pushed, since the reverse reaction will be favoured under certain conditions.

Under anaerobic conditions, *C. necator* is capable of denitrification, in which it uses oxidized nitrogen compounds as terminal electron acceptors instead of O_2_ [14]. To do so, it expresses genes encoding reductases for nitrate, nitrite, nitric oxide, and nitrous oxide [14, 35]. These enzymes give the option of culturing *C. necator* in waste streams that do not contain oxygen, which widens the range of applications for which it could be used. However, the growth rate with nitrate as the terminal electron acceptor was reduced by 90–95% relative to growth with O_2_ as the electron acceptor [36]. Alternative denitrifying chassis could be considered, but for chemical production, it is likely preferable to simply operate with a better electron acceptor than nitrate.

All this cellular machinery allows *C. necator* to use different carbon and electrons sources, and electron acceptors. This affords it metabolic flexibility, making it a generalist that can survive in a variety of conditions, including environments with low nutrient availability. Under such conditions, it is also capable of storing excess carbon in the form of poly-3-hydroxybutyrate (PHB) granules in its cytoplasm [11, 17]. PHB synthesis is induced when nitrogen, oxygen, or phosphorus is limiting, but nitrogen-limitation leads to the highest accumulation of PHB [37, 38]. Production of PHB itself is important because it is a biodegradable polymer used in many applications in industry and medicine [13, 39]. However, the pathway for its production is also relevant to biomanufacturing because it offers several nodes from which alternative pathways can be integrated to produce other value-added compounds. The precursor to PHB production is acetyl-CoA, which is used in a number of endogenous and exogenous pathways and can therefore be utilised for production of a wide range of chemicals. The intermediates between acetyl-CoA and PHB are acetoacetyl-CoA and 3-hydroxybutyryl-CoA, and each of these molecules can also be directed towards other pathways.

Although its metabolic versatility makes *C. necator* interesting for biomanufacturing, this adaptability comes at an efficiency cost that impedes the optimisation of characteristics required for industrial use. *C. necator* maintains a large unutilised fraction of its proteome to maximise its environmental readiness [40], but this increased protein production is a burden on growth and production. The complexity of *C. necator* metabolism and proteome means rational engineering must consider many factors and should be physiology-informed. Designing a *C. necator* strain for the purpose of biomanufacturing may require substantial genomic modifications to shift it from a generalist to a high-producing specialist. Therefore, genome minimization—or at least reduction—is a likely requirement for designing an industrial *C. necator* strain.

**Fig. 1:**
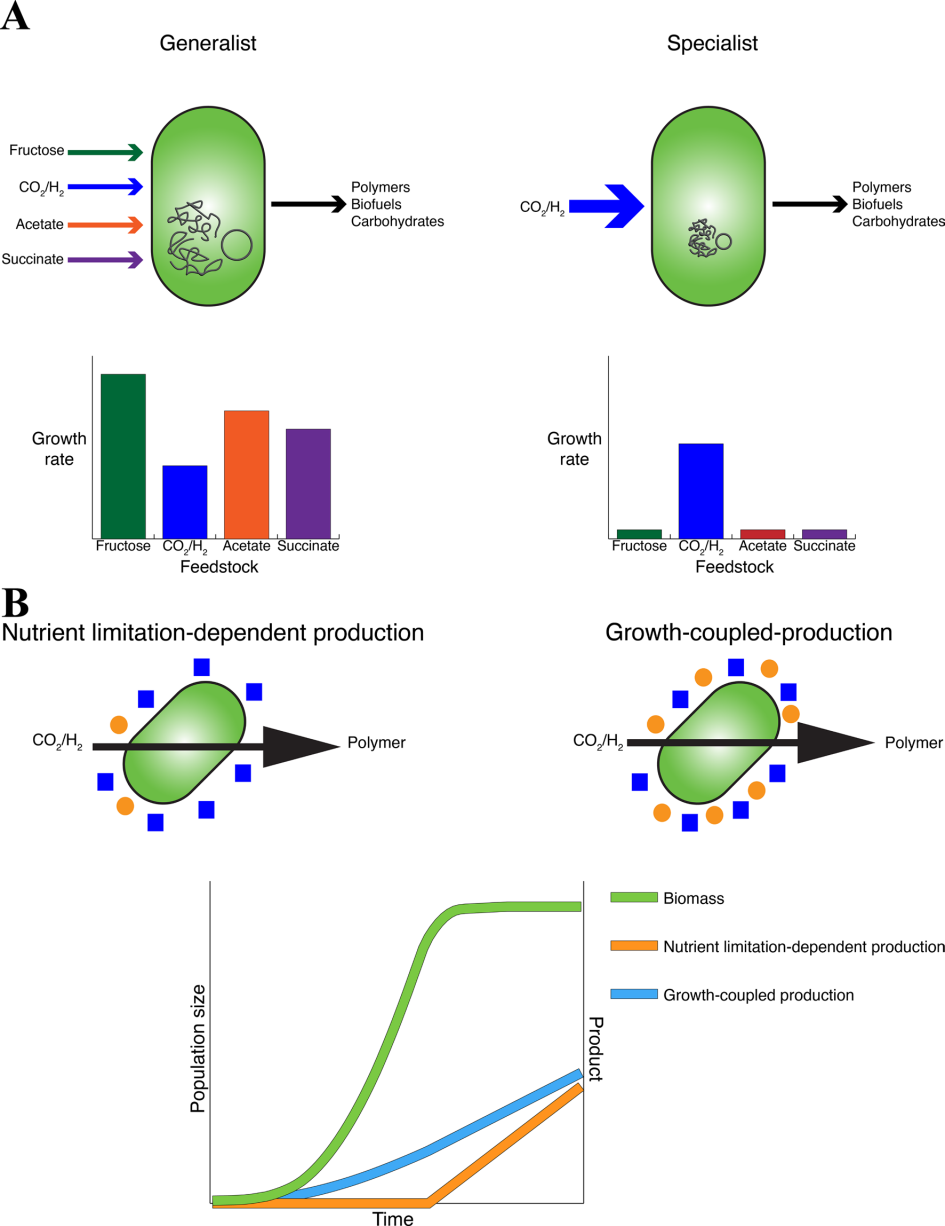
Strategies for engineering an industrially relevant *C. necator* platform. **A** Changing *C. necator* from a generalist to a specialist involves genome reduction to eliminate genomic redundancy and unneeded assimilation pathways. This will result in increased growth rate on the chosen substrate and decreased growth rate on all other substrates. **B** Achieving growth-coupled production may involve modifying the stringent response. For nutrient limitation-dependent production, production is delayed but more efficient during stationary phase. For growth-coupled production, production is slower during stationary phase, but production happens throughout the growth cycle. Note that the graphs are schematics and do not correspond to true values

Jahn et al. found that deletion of certain hydrogenase-related genes increased growth rate in heterotrophic conditions by reducing the burden of producing under-utilized proteins [41]. Similarly, Calvey et al. showed that deletion of the transcriptional regulator *PhcA* and the pHG1 megaplasmid improved growth on formate [42]. These results support the idea that deletion of genes for alternative growth conditions can help improve performance under specific growth conditions (Fig. 1). This would potentially mean developing *C. necator* strains for each of its possible substrates, with each strain being optimised for production of valuable chemicals from the chosen feedstock. Jahn et al. also identified that most of the formate oxidation was due to the soluble formate dehydrogenase rather than the membrane-bound formate dehydrogenase [41]. This result suggests that reducing redundancy of the genome could have little negative effect on metabolic flux, while reducing the protein burden. Certain deletions of redundancy would likely be relevant to all strains of *C. necator*, while others may be undesirable for given growth conditions.

Another necessary step in making *C. necator* an effective industrial chassis is to decouple PHB production from nutrient limitation (Fig. 1). PHB production is natively induced by limited availability of nitrogen, oxygen, or phosphorus [37, 38]. However, for biomanufacturing purposes, it is expected that production in parallel with balanced and continuous growth would simplify fermentation processes and improve production [38, 43–45]. Sander et al. modelled this approach with an engineered *Cupriavidus basilensis* 4G11 strain exhibiting growth-coupled production and concluded that a 24% increase in productivity was achievable compared to the wild-type strain under optimised conditions. However, growth-coupled PHB production has yet to reach the accumulation achieved during nutrient limitation [43]. The sigma factor σ-54 encoded by *rpoN* is involved in regulating nitrogen metabolism, and it has been identified as a potential target for decoupling production from nitrogen limitation. In fact, its overexpression led to increases of 54.4% and 103.1% for PHB content in the cell and PHB titre, respectively, under heterotrophic conditions, as well as 44.1% and 77.5% for autotrophic conditions [46]. Given that limitation of other nutrients also results in production of PHB, the overall stress response of *C. necator* is also worth considering. The stringent response, a mechanism present in many organisms to protect against stress caused by nutrient limitation, is controlled by nucleotide guanosine tetraphosphate (ppGpp), which affects the sigma factor σ-70, which in turn affects σ-54. Chemical induction of the stringent response resulted in increased PHB accumulation under non-stressed conditions [47].

Controlling the stringent response is also applicable to products other than PHB. Salinas et al. found that for heterologous 3-hydroxypropionic acid (3-HP) production in *C. necator*, flux toward 3-HP was increased under nitrogen limitation [48]. This suggests that the stringent response is a target for increasing flux toward PHB and toward heterologous pathways. Salinas et al. also observed that triggering the stringent response later, after more biomass had been accumulated, resulted in a greater titre of 3-HP in a strain unable to produce PHB. Growth-coupled production would avoid the need to induce at an optimal time. It was also seen that PHB co-production had a favourable effect on 3-HP synthesis, likely because the PHB pathway consumes NADPH, and the TCA cycle replenishes it while producing the intermediates for 3-HP [48]. To eliminate the need for PHB co-production, a feedstock assimilation pathway that balances cofactors with the product synthesis pathway could be implemented. In this mixotrophic approach, where autotrophy is supplemented with heterotrophy, CO_2_/H_2_ and the other feedstock would be co-consumed [49, 50]. This design would avoid directing carbon flux toward making PHB, while maintaining the driving force for desired production pathways. Furthermore, removing PHB co-production would make modifying the stringent response a potential strategy for increasing product synthesis.

Thus, to make *C. necator* a high-producing specialist, its genome should be reduced, and the PHB production pathway should be decoupled from nutrient limitation. These strategies and their effects are summarized in Fig. 1. Genome reduction would alleviate the burden of maintaining many unused and/or redundant proteins, as this feature is useful for a generalist microbe that consumes feedstocks infrequently used by other organisms, but it is not relevant to a specialised industrial chassis. Similarly, production of PHB under nutrient limitation is useful for an organism undergoing varying levels of nutrient availability, but in a controlled environment, decoupling production of PHB or of other chemicals from nutrient limitation would allow simpler processes and increased productivity.

### Improving autotrophic metabolism

Many of the current attempts to use *C. necator* for biomanufacturing have focused on PHB production, for which the pathway is endogenous, because it simplifies the genetic modifications required [51–55]. Focus can thus be on engineering substrate consumption and flux control rather than the integration of an exogenous pathway. However, the potential for fixing CO_2_ to produce chemicals extends beyond PHB. Biofuels are also of great interest because their production from CO_2_ would further help overcome societal reliance on fossil fuels, notably in the transport industry. *C. necator* has been used to produce biofuels such as alcohols, fatty acids, ketones, alkanes, and terpenoids [56]. *C. necator* has also been used to produce carbohydrates like sucrose and lipochitooligosaccharides [57]. While engineering the bacteria to produce these compounds of interest and others is an achievement, the cells must be engineered to meet industrial production requirements. For this purpose, *C. necator* fermentation can be combined with an electrochemical system or used on its own. In both cases, there is a need to increase the focus on using and improving the native autotrophic metabolism of *C. necator*.

#### Microbial electrosynthesis

Microbial electrosynthesis (MES) is an approach that combines electrosynthesis, in which catalysts powered by electrical energy drive chemical reactions, with microbes that act as CO_2_-reducing biocatalysts, or to perform other redox reactions using organic feedstocks [58, 59]. In this system, electricity provides electrons directly or indirectly to the microbe. With this acquired reducing power, the microbe can catalyse reaction(s) such as CO_2_ reduction [58–60]. Artificial leaf systems use this same principle but harvest light energy to generate an electric current, thus mimicking a photosynthetic system; however, the artificial leaf can achieve greater efficiency than natural photosynthesis [17]. *C. necator* is a useful organism for MES because it naturally consumes H_2_, an effective electron carrier for energy transfer from the electrodes to the microbes, so relatively little work is required to allow *C. necator* to grow in an electrolytic cell.

In an artificial leaf system with an engineered strain of *C. necator*, the bacteria grew at a solar-to-biomass yield up to 3.2% and produced 216 mg/L of isopropanol at a 90% yield. However, toxic reactive oxygen species were generated at low potential, limiting the system effectiveness. Resolving this issue required managing the potential to balance water splitting and ROS generation, as well as sparging with CO_2_ and adding catalase to improve cell survival [61].

Krieg et al. used a standard MES system to produce up to 10.8 mg/L α-humulene in 7 days using an engineered *C. necator* strain. Like the artificial leaf system, cell viability was reduced by the presence of toxic ROS [62]. To overcome this issue, Chen et al. used a proton exchange membrane (PEM) to prevent ROS produced at the anode from reaching the cells at the cathode. The authors also used formate produced from CO_2_ using a formate dehydrogenase to deliver electrons to the cells, electron carriers to transfer electrons from the cathode to formate, and overexpression of the RuBisCo from *Synechococcus elongatus* PCC7942, to achieve 485 mg/L PHB produced in 5 days [52]. However, the electron transfer system using the carriers appeared not to transfer all electrons, as the reduced form of one of the electron carriers used, neutral red, was present at the end of the fermentation. The addition of these electron carriers may therefore be an overcomplication when electrochemical reduction of CO_2_ to formate is possible [63]. However, the potentials required for electrochemical reduction of CO_2_ to formate may not be optimal for the rest of the system, further reinforcing the challenges that come with MES.

Another way to mitigate the effect of ROS generation is to produce an antioxidant. Wu et al. produced 1.73 mg/L of lycopene, a powerful antioxidant, in 4 days using exhaust gas from a coal-fired power plant. The lycopene-producing strain tolerated electrolysis better than the control strain because the cells were protected from ROS by lycopene [28]. A potential pitfall to this strategy is that it limits the applications of MES to products that are antioxidants, or it requires that an antioxidant be produced as a side product, which increases the metabolic burden imposed on the chassis. If products must be chosen based on the method, then MES will not be broadly relevant.

To overcome both potential management and ROS toxicity, Lim et al. used physical separation: the fermenter and CO_2_ electrolysis were in separate chambers, and the cathode and catholyte were separated from the anode and anolyte. The catholyte contained formate produced at the cathode but was separated from the anolyte containing ROS by a Nafion ion exchange membrane. The catholyte flowed from the CO_2_ electrolysis chamber to the fermenter and was designed to also act as the culture medium. This MES system resulted in 1.38 g of PHB in 5 days, with the cells having accumulated PHB being removed from the culture and fresh cells being added [53]. However, it is possible that using one solution for both electrolysis and fermentation is limiting, because it cannot be optimized for both tasks.

Another option—unexplored for MES—to overcome ROS toxicity would be to use a hydrogen peroxide tolerant strain of *C. necator*. One such strain was developed using a CRISPRi-Mutator technology, the mutations from which increased the expression of superoxide dismutase and catalase [64]. Nonetheless, despite the multiple strategies to mitigate the inherent issues of MES, further optimisation of both the electrolysis and fermentation processes is necessary because the titre, productivity, and yield values obtained so far are below the requirements for industrial production [65, 66]. It is worth considering that decoupling the electrochemical and microbial components would allow each one to be completely optimised and thus improve the overall process [66]. A technoeconomic assessment would be beneficial to direct future efforts in this field. Nonetheless, for MES or for a decoupled solution, further optimisation of the microbial fermentation is necessary. Utilizing and improving the natural abilities of *C. necator* could be the path towards industrial use of this technology.

#### Heterotrophic fermentation

In addition to MES and decoupled electrochemical and microbial systems, the ability of *C. necator* to use CO_2_ makes gas fermentation a potential strategy for sustainably producing value-added products. Either way, if fermentation is coupled to another strategy or if it is used alone, improving the carbon assimilation and chemical production of *C. necator* will be necessary. Improving fermentation conditions is one area of interest: Nygaard et al. showed that fed-batch fermentation with exponential feeding of fructose allowed the process to be scaled to a 5 L bioreactor with a PHB productivity greater by 3.3-fold and 7.2-fold than batch and shaken flask fermentation strategies, respectively [54]. However, as this study involved heterotrophic *C. necator* growth, its impact is limited for many sustainable applications. Nonetheless, it demonstrates the importance of tuning fermentation parameters when optimising production. That being said, process engineering is not likely the bottleneck in reaching industrial production from *C. necator* fermentation. As long as the process allows sufficient growth, metabolic engineering could have a more significant impact on production because it can help control the direction of flux and can allow production of exogenous products. Therefore, much work to improve the output of microbial fermentation has focused on strain engineering.

Other heterotrophic growth studies have focused on producing compounds of interest from a variety of feedstocks. Importantly, varying the feedstock can help control the product: polyhydroxyalkanoates (PHAs) containing specific 2-hydroxyacid monomers—2-hydroxybutyrate (2HB), 3-hydroxybutyrate (3HB), 3-hydroxyvalerate (3HV), and lactic acid (LA)—were produced by feeding 2-hydroxybutyrate, glucose, or lactate [67]. Thus, while heterotrophic growth does not take advantage of the autotrophic capacity of *C. necator*, it is useful to obtain specific monomers resulting from the breakdown of the chosen substrate. Beyond providing specific molecules, heterotrophic growth, typically on fructose, has been used to develop engineered strains before shifting to autotrophic growth. For example, Cre´pin et al. developed an alka(e)ne-producing strain that resulted in 4.3 g/L of total alka(e)nes when consuming fructose. Subsequently, they used the same engineered strain and fed it CO_2_ as the sole carbon source for production, and this resulted in 4.4 mg/L of total alka(e)nes. It is worth noting that in the autrotrophic case, fructose was still used during a growth phase that preceded the production phase [68].

#### Autotrophic fermentation

For the purpose of engineering of autotrophic metabolism, growth on both CO_2_/H_2_ and formate will be considered. Growth on formate is organolithotrophic, but since the formate dehydrogenase of *C. necator* converts formate to CO_2_, the metabolic pathways used subsequently to assimilate formate are those used during autotrophic growth. Although *C. necator* naturally consumes these substrates, their use for biomanufacturing has motivated many studies aiming to improve carbon fixation. As the natural carbon fixation pathway for *C. necator*, the CBB cycle is the main target for improvements.

Rather than improving the CBB cycle, which has been seen as inefficient, Claassens et al. implemented an alternative carbon-fixing pathway. The reductive glycine pathway (rGlyP) converts formate to glycine—and thus, eventually, biomass—in a more ATP-efficient way than the CBB cycle that is predicted to lead to increased biomass, pyruvate, and acetyl-CoA [69, 70]. Increased growth and production of important metabolic intermediates such as pyruvate and acetyl-CoA would help make *C. necator* a more useful biomanufacturing chassis. This pathway resulted in a growth yield of 2.6 gCDW/mole-formate, slightly lower than the growth yield of 2.9 gCDW/mole-formate of the wild-type strain using the CBB cycle. The authors expect that further optimisation would allow the rGlyP-using strain to surpass the wild-type strain [69]. However, though the CBB cycle has been understood as inferior to other carbon-fixing pathways, more recent analyses have shown that the enzymes in the CBB cycle, other than RuBisCO, are more active and their thermodynamics and kinetics are more favourable than the enzymes in these other pathways [71]. Therefore, it may be preferable to engineer the native CBB cycle instead of replacing it.

Overexpression of the *Synechocystis* sp. PCC6803 genes encoding RuBisCO, *rbcL* and *rbcS*, and the assembling chaperone, *rbcX*, increased the growth rate of *C. necator* compared to its endogenous RuBisCO. In addition, overexpression of the transcriptional regulators *cbbR* and *regA*, which are key to controlling expression of the CBB pathway genes, resulted in an 11% increase in biomass accumulation and a 28% increase in PHB production for autotrophic growth of *C. necator* on CO_2_ [55]. Similarly, another study showed increased growth when the *Synechococcus* sp. PCC 7002 RuBisCO genes and the endogenous *C. necator* chaperone genes *groES/groEL* were overexpressed [51]. The authors combined this approach with upregulation of operons encoding the MBH and SH. The resulting strain achieved increases of 93.4% and 74.7% for growth and PHB production, respectively, compared to the control strain [51].

Beyond engineering RuBisCO, adaptive laboratory evolution (ALE) and transcriptomic data were used to identify mutations of interest for improving growth on formate [42]. This led to a strain lacking the transcriptional regulator *PhcA* and the pHG1 megaplasmid that achieved a 24% increase in maximum growth rate on formate, as well as 7% and 19% increases for growth on succinate and fructose, respectively [42]. These results further suggest that reducing the genome and thus reducing the generality of *C. necator* may be a valuable strategy for improving growth on a specific substrate, as was discussed in Sect. "Shifting from generalist to specialist".

With improvements to the carbon fixing ability of *C. necator*, production of chemicals of interest becomes more feasible. Production can be shifted away from PHB and towards these valuable chemicals. A study by Windhorst et al. redirected flux from PHB to acetoin, a platform chemical, by deleting the *phaC1* and *phaC2* genes encoding PHA synthases and by expressing the acetoin pathway that branches off from the endogenously produced pyruvate. The authors also deleted the operon encoding the *acoABC* genes responsible for acetoin consumption [72]. The combination of these genetic changes resulted in the autotrophic production of 13.66 mM of acetoin in 70 h at a yield that exceeded the theoretical maximum, due to cell lysis according to the authors [72].

Gascoyne et al. produced up to 33 mM of 1,3-butanediol (1,3-BDO) from CO_2_ in 84 h, with an average yield of 40% of the theoretical maximum. As with the production of acetoin, pyruvate is a key metabolite, since 1,3-BDO production requires 3-hydroxybutyraldehyde-CoA (3HBCoA), an intermediate in the pathway leading from pyruvate to PHB. Taking advantage of this well-known pathway in *C. necator*, the authors overexpressed the *phaA* and *phaB* genes to produce more 3HBCoA, and they deleted the *phaC1* gene to prevent this intermediate from being converted to PHB. Instead, the flux was directed to 1,3-BDO via the *bld* gene for butanal dehydrogenase and the *YqhD* gene for aldehyde reductase [73]. Collas et al. produced 148 mg/L of crotonate from formate at a yield of 1.70 mg_*Crotonate*_/g_*Formate*_ in 72 h [74]. Similar to the production of 1,3-BDO, the node of interest is 3HBCoA, as crotonyl-CoA, and then, crotonate can be produced from this intermediate. While the endogenous pathway for producing 3HBCoA could be used, the authors achieved greater production of crotonate by using alternative engineered routes and knocking out the *phaA*, *phaB*, and *phaC* genes [74].

Wang et al. produced 105 mg/L of isoleucine from CO_2_ by circumventing the long native pathway for isoleucine with a shorter exogenous one. The authors also engineered a strain to produce 319 mg/L of valine from CO_2_ by overexpressing genes of interest. In both the isoleucine- and valine-overproducing strains, feedback regulation limits the overproduction of amino acids to maintain balance in the cell, so feedback-resistant versions of key enzymes were expressed. Furthermore, in both strains, the phaC1AB1 operon was knocked out, similarly to previous strategies. However, to prevent the accumulation of the NADPH that would normally be consumed during the production of PHB, the authors expressed NADPH-dependent versions of key enzymes rather than NADH-dependent ones [75]. This strategy for cofactor balancing should be considered and implemented when possible, as deletion of the PHB-producing pathway is a common modification for increasing production of value-added chemicals. Alternatively, PHB synthesis can be utilised as a sink for excess electrons, as was done in *Herbaspirillum seropedicae* [76].

Another example of this PHB pathway deletion approach was in the production of 1.9 mg/L of resveratrol from CO_2_ and tyrosine, in which the authors also expressed three heterologous genes allowing the conversion of tyrosine to reseveratrol, and overexpressed the gene encodingacetyl-CoA carboxylase to provide more malonyl-CoA needed to produce resveratrol [77]. While supplementation of tyrosine or *p*-coumaric acid has been used in other resveratrol productions [78, 79], to make this process more reliant on CO_2_, it would be relevant to engineer the strain to increase its natural production of tyrosine. In addition, much work is still necessary to achieve industrial production of all these value-added compounds.

For the purpose of using *C. necator* in biomanufacturing, certain feedstocks should be prioritized. *C. necator* is unable to naturally consume glucose. Nonetheless, Wang et al. engineered it to consume glucose for production of isoleucine and valine [75]. Many other chassis naturally consume glucose and produce isoleucine and valine. Furthermore, glucose and other sugar feedstocks are typically sourced from agricultural products, so using them for biomanufacuring competes with food production and encourages unsustainable practices associated with industrial agriculture [80]. Thus, although achieving isoleucine and valine production from glucose demonstrates the progress that has been made in our ability to engineer *C. necator*, from a biomanufacturing point of view, it may be preferable to focus on designing *C. necator* strains that consume sustainable feedstocks, or at the very least, save time by using the endogenous fructose-consuming pathway of *C. necator*.

**Fig. 2:**
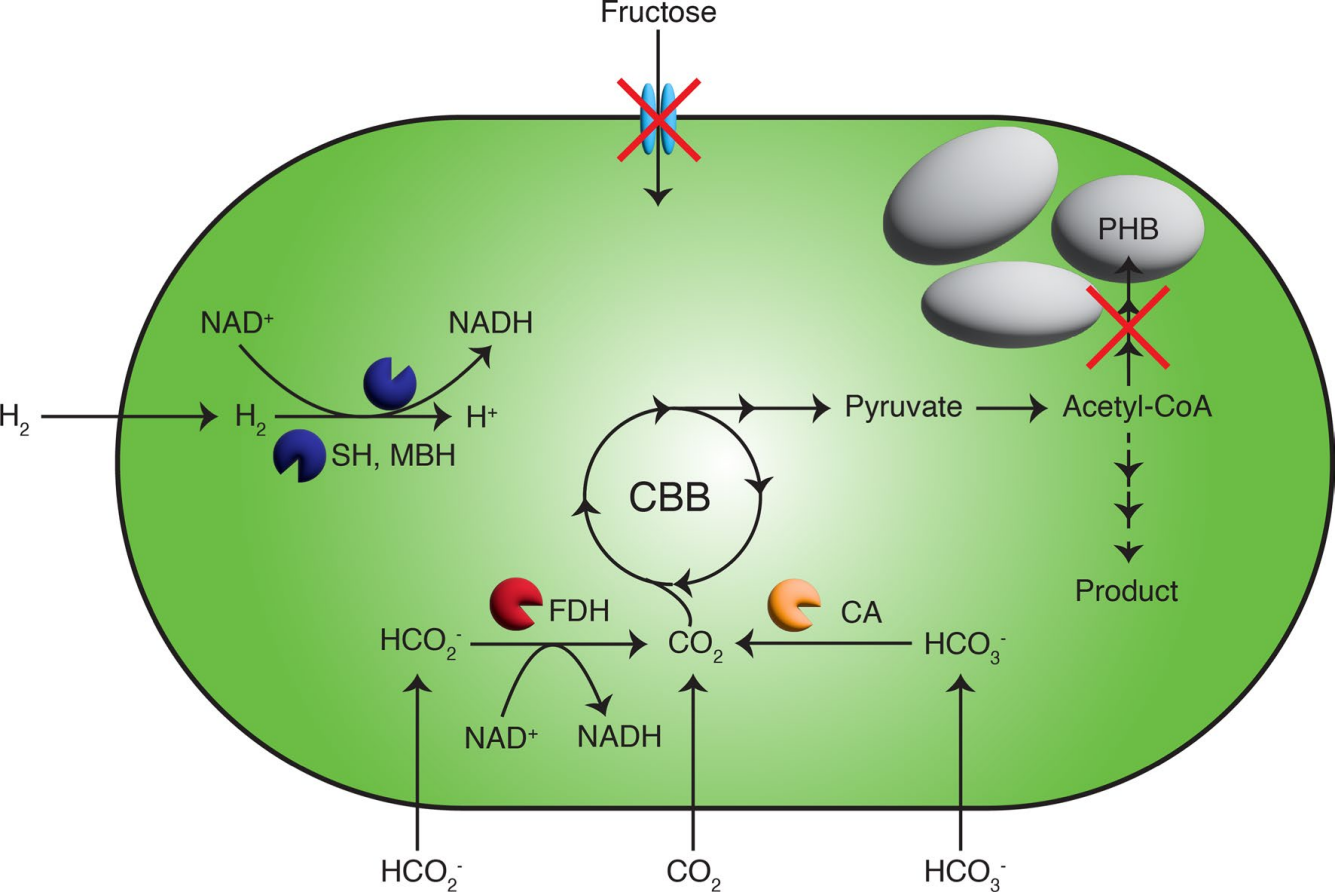
Schematic of the operation of *C. necator* for optimal sustainable production. Autotrophic metabolism, including growth on CO_2_/H_2_ and on formate, should be prioritised to make this technology sustainable. Similarly, sugar feedstocks like fructose should be avoided. Eliminating PHB production will allow carbon to be diverted toward production of chemicals of interest. *PHB* poly-3-hydroxybutyrate, *SH* soluble hydrogenase, *MBH* membrane-bound hydrogenase, *FDH* formate dehydrogenase, *CA* carbonic anhydrase-like enzymes

However, if the goal is to eventually use sustainable substrates, then the engineering efforts should focus on this from the start. Many studies started by engineering heterotrophic production of a chemical of interest, and followed this by simply growing the engineered strain autotrophically [68, 72, 73, 75, 77]. Since a large fraction of *C. necator*’s proteome is unused, shifting between autotrophic and heterotrophic growth may be prohibitively slow using this strategy [40]. Given that autrotrophic growth and production is already established, this approach could be used from the start, and all engineering efforts would thus be contributing to the final design. This operation of *C. necator* in this approach is presented in Fig. 2.

Engineering strategies focusing on improving autotrophic metabolism should aim to increase CO_2_ fixation, as has been done already, but also to overcome the fundamental limitation of CO_2_ as a feedstock: it is not a source of energy. H_2_ is a source of electrons that *C. necator* can use, but additional reducing power could improve the performance of the autotrophic metabolism and of carbon fixation, as it would provide the power necessary to reduce CO_2_. In addition, Manoli et al. showed that co-consumption of substrates could improve PHB productivity in *Pseudomonas putida* [45], so this strategy would potentially also be useful in *C. necator*. Therefore, future use of *C. necator* should take advantage of its autotrophic growth, and engineering strategies should focus on improving autotrophic metabolism of *C. necator*.

### Designing reliable tools

Engineering *C. necator* for improved growth and production of such diverse chemicals has been made possible thanks to the development of chassis-specific tools. Similarly, sequencing of the *C. necator* genome and subsequent modelling have made it much easier to efficiently predict the effect of engineering strategies rather than using time-consuming iterative experimental approaches. Available tools for *C. necator* are presented in Table 1. Despite the progress, there is still much work to be done in order to make engineering *C. necator* even more straightforward and effective.

**Table 1:** Available tools for engineering *C. necator*

Tool	Use	Purpose	References
Bioconjugation	Experimental	Transformation	[81]
Chemical transformation	Experimental	Transformation	[82]
Electroporation	Experimental	Transformation	[81, 82, 84]
pCAT vector series	Experimental	Plasmid	[82]
pMTL70000 vector series	Experimental	Plasmid	[85]
Tn5 transposon	Experimental	Genome engineering (random integration)	[56]
RalstTron	Experimental	Genome engineering (target gene knockout)	[56]
Homologous recombination + suicide plasmid	Experimental	Genome engineering (targeted gene knockout)	[56]
CRISPR-Cas9	Experimental	Genome engineering	[84]
CRAGE	Experimental	Genome engineering	[20, 91]
CIFR	Experimental	Genome engineering	[92]
Constitutive promoters	Experimental	Regulation	[93, 94]
RBSs	Experimental	Regulation	[64]
CRISPRi	Experimental	Regulation	[64]
ALE	Experimental	Controlled mutation	[42, 64]
Sequenced genome	Computational	Identifying genetic features	[30]
GSM	Computational	FBA, RBA	[40, 100, 103]

#### Synthetic biology tools

##### Transformation

Transformation is an essential step in synthetic biology and metabolic engineering, as it allows foreign DNA to be introduced into a chassis. For *C. necator*, the standard method was conjugation until recently. Conjugation is the process of introducing the recombinant DNA into a donor strain of a different species that is more easily transformed and then transferring the DNA to the recipient strain, *C. necator*, by mating. The successive transformation and culturing steps make this process time and resource intensive [81]. Therefore, to make engineering *C. necator* more feasible and attractive, a more efficient and effective transformation method was required.

Chemical transformation is a common method for model organisms, as it is relatively simple and does not require specialized equipment. In *C. necator*, transformation efficiency of approximately 250 cfu/μg DNA was achieved by Azubuike et al. However, this transformation required a large quantity of DNA (1 μg) [82]. A chemico-physical transformation protocol was also developed—in which 0.1 M RbCl was used to increase permeability of the membrane, and 0.1 mg/ml gold(III) chloride was used to pierce the membrane—and achieved 3.94×10^4^ cfu/μg DNA [83]. These transformation efficiencies still need to be improved for productive use in metabolic engineering.

While electroporation is generally known to be an effective transformation method, in *C. necator*, transformation efficiency was previously very low (10^2^-10^3^ cfu/μg DNA) [56]. Tee et al. improved the electroporation conditions and achieved an efficiency of 3.86×10^5^ cfu/μg DNA [81]. Electroporation efficiency was also reported to be low due to the restriction modification (RM) system of *C. necator*: a study found that deleting the RM genes *H16 A0006* and *H16 A0008-9* resulted in 1658- and 4-fold greater electroporation efficiencies, respectively, and 1697-fold when both were deleted [84]. However, these deletions were repeated in a later study and only showed a 3- and 1.3-fold increase, respectively, and 16-fold increase for deletion of both [85]. These studies used different plasmids, so the resulting transformation efficiencies were drastically different (100 and 30,000 cfu/μg DNA) [84, 85]. Another study showed that certain features of broad host range (BHR) plasmids typically used in *C. necator* were responsible for reducing electroporation efficiency [82]. With improved plasmid design, this study increased electroporation efficiency by over 3000-fold compared to the standard control plasmids and reached efficiency up to 10^7^ cfu/μg DNA [82]. Thus, the comparison between the two studies deleting the RM genes may not be fair since efficiency in either study might have been affected by the plasmids used. This potential issue highlights the need to standardize the plasmids used to engineer *C. necator*.

##### Plasmids

The most commonly used plasmids to engineer *C. necator* are BHR plasmids—which can transfer, replicate, and be maintained in many microorganisms across phylogenic subgroups [56, 86]. The BHR plasmids include pCM, pCM271, pBBR1, pKT230, pSa, pCUP3, and pBHR1 [56, 82, 87–90]. These plasmids lack the standardization and modularization that are foundational to synthetic biology. In addition, they are not specifically designed for *C. necator* or for use in high-throughput and parallel plasmid assemblies, and they result in poor transformation efficiency and stability [82]. The pCAT and pMTL70000 vector series plasmids were designed to overcome these issues [82, 85]. The pCAT vectors are modular designs that include a replication module, an antimicrobial resistance module, and an application-specific module that can include a reporter gene or a multiple cloning site (MCS) [82]. They use a version of the kanamycin resistance gene that does not impede electroporation, as the version in pBBR1 does, and use a complete copy of the replication module to improve stability of the plasmid [82]. The pMTL70000 vectors are also modular: they include a *C. necator* replicon module, a selectable marker module, an *Escherichia coli* replicon module, and an application-specific module. The plasmids in this series are well-characterized in terms of transmissibility, stability, copy number, and compatibility [85].

##### Genome engineering

Genome engineering is crucial for strain design, as it is needed to knock in genes for stable integration and to knock out genes for gene deletion. Therefore, the existence of a wide range of genome engineering tools for a given chassis makes that chassis easier to use. For *C. necator*, Tn5 transposon is a tool for random integration, RalsTron is a tool for targeted gene knock-out using group II introns, and homologous recombination, often with suicide plasmids to improve efficiency, is used for gene disruption and insertion [56]. However, poor efficiencies and slow procedures limit these techniques. For most chassis, the commonly used genome engineering techniques involve the CRISPR-Cas9 system. Xiong et al. developed a CRISPR-Cas9 technique in which the *cas9* gene is expressed under an arabinose-inducible pBAD promoter. Using an induction time of 168 h, this method resulted in editing efficiencies ranging from 78.3 to 100% [84]. However, integration of large DNA sequences is still a challenge and will therefore require further research [56].

Chassis-independent recombinase-assisted genome engineering (CRAGE), developed by Wang et al., is intended to deliver large biosynthetic gene clusters (BGC) into a wide range of hosts. The method works by conjugating a first plasmid into the host, where a landing pad is integrated into the genome by a transposase, followed by a second plasmid containing the BGC, which is integrated using a recombinase and lox sites on the landing pad and around the BGC [91]. Panich et al. modified this method to be more effective in *C. necator*, and they successfully integrated the expression cassette for a dissolved inorganic carbon transporter [20].

Iterative genome engineering steps can also be a challenge due to the limited number of available selection markers. Federici et al. developed a method based on Tn5 integration that allows antibiotic selection markers to be excised and subsequently reused. The method, named CIFR (Clone-Integrate–Flip-out–Repeat), involves flanking the antibiotic resistance gene with recombination sites for the Bxb1 integrase, which is expressed on a second plasmid that contains an alternative antibiotic resistance gene and the *sacB* gene for counter-selection and curing of the plasmid. The authors successfully applied the method in *C. necator*, achieving 95% efficiency in the removal of the integrated antibiotic resistance gene [92]. For iterative targeted genome engineering, it would be of interest to combine this workflow with an integration technique such as CRISPR-Cas9 instead of random Tn5 integration.

##### Regulation

Promoters and ribosome binding sites

Inducible promoters, such as the P_*BAD*_ and P_*tet*_ promoters that were previously typical for use in *C. necator*, are limited for use in industrial applications because expressing multiple genes would require more inducible promoters than are available. Also, with multiple inducers comes the potential for crosstalk. Furthermore, providing inducer at such a large scale is economically unfeasible [93].

Therefore, since the use of *C. necator* for biomanufacturing implies that the technology must be scalable, engineering constitutive promoters is more relevant.

Johnson et al. expanded the expression range from 6-fold difference for the most common constitutive promoters for *C. necator*—P_*phaC1*_, P_*rrsC*_, P_*j5*_, and P_*g25*_—to 137-fold difference for a library of 42 constitutive promoters derived from the standard ones [93]. For the purpose of the study, the authors included the RBS in the definition of their promoters, so explicit engineering of the RBS could be a useful tool for more extensively controlling expression. In a different study, Alagesan et al. developed a library of 29 constitutive promoters with a range of over 700-fold difference in activity. The authors also created a library of 27 RBS sequences with a range of over 10-fold difference in activity. A library of RBSs can be useful to modulate gene expression differently for different genes in the same operon. However, it is important to note that the context around the sequence affects its absolute activity, so RBS sequences should be evaluated on a case-by-case basis [94].

CRIPSRi

To make use of the high specificity of the CRISPR gene editing system, CRISPRi uses a catalytically inactive version of the Cas9 enzyme (dCas9), which allows the target sequence to be bound without being cut. This binding blocks the transcription elongation of the sequence and results in repression of the target gene [95]. Using the CRISPR system developed by Xiong et al., Wang et al. developed a CRISPRi system for *C. necator*. This system constitutively expressed the sgRNA and inducibly expressed a codon-optimised dCas9. The authors showed that the design of the sgRNA and the amount of inducer (arabinose) can be used to control the level of repression caused by the CRISPRi system. They demonstrated this repression ability for an *rfp* reporter gene and for the native *phaC* gene [64]. While knockdown of genes can be useful to avoid disrupting intracellular metabolic balance, the metabolic burden of the CRISPRi system makes knockout preferable for cases where a gene is not essential and not needed. Therefore, the value of the CRISPRi system may lie in other applications like facilitating ALE.

##### Adaptive laboratory evolution

ALE is the process of culturing cells in a controlled and targeted environment for an extended period of time and selecting for the most beneficial naturally acquired mutations [96]. This technique is valuable to achieve improvements in properties for which researchers do not necessarily know the genetic cause. Calvey et al. identified such random mutations by growing *C. necator* on minimal media with formate as the sole source of carbon and energy for 400 generations [42]. While this method has proven to be successful in *C. necator*, slow spontaneous mutation rates of many bacteria prevent the fast and effective application of ALE. To overcome this limitation, Wang et al. used their *C. necator* CRISPRi system to develop a CRISPRi-Mutator for rapid and inducible genome evolution. They targeted the *mutS* gene, involved in DNA mismatch repair, with the CRISPRi system to allow a strain to temporarily mutate faster while the system is induced but to return to normal function for further engineering and characterization after the ALE. They successfully demonstrated the acquisition of chloramphenicol resistance, isopropanol and isobutanol tolerance, and hydrogen peroxide resistance in separate experiments using the CRISPRi-Mutator [64].

#### Modelling tools

Modelling *C. necator*, or any other chassis, can help predict the effects of genetic changes on the metabolism of the organism. These results can then help inform and plan experiments, thus accelerating metabolic engineering designs and saving on laboratory consumables. However, this is reliant on the availability and quality of computational tools. To use non-traditional chassis for biomanufacturing, it is imperative that such tools be developed quickly and accurately to help meet the pressing demands for a shift in the chemical industry. In the case of *C. necator*, progress has and is being made, but more work is still required.

##### Genome-scale models

A genome-scale metabolic model (GSM) is an important tool for modelling: it represents the metabolic network of the organism through the stoichiometry of the metabolic reactions identified from the genome sequence. Thus, it can be used to predict the metabolic states that could be reached under given conditions or following given genetic modifications [97, 98]. Constructing a GSM requires a sequenced genome in which the enzyme-coding sequences are predicted and identified [98]. The peptides and proteins are then mapped onto these enzyme-coding regions, and the reactions catalyzed by these proteins and their metabolites are then associated to the genes. In the absence of complete proteomics and metabolomics data, which is the case for most organisms, template models are used as comparisons to help identify the missing reactions, and multiple iterations help improve the number of reactions included in the GSM [98, 99].

The genome of *C. necactor* H16 was published in 2006 [30]. This sequence was used to annotate reactions for the Kyoto Encyclopedia of Genes and Genomes (KEGG) database, which was then used to develop the first published GSM of *C. necator* H16: RehMBEL1391 [100, 101]. This GSM contained 1391 reactions and 1171 metabolites [100, 101]. Lack of stable reaction and metabolite IDs and lack of computer readability of model files made this GSM difficult to update [101, 102]. However, it was eventually updated to improve the ID naming and to add annotations. This updated GSM had 1538 reactions and 1172 metabolites [40, 101].

The second published GSM of *C. necator* H16, *i*CN1361, was developed using the BioCyc database and has 1292 reactions and 1263 metabolites [101, 103]. *i*CN1361 has better stoichiometric consistency and reaction balance than RehMBEL1391, which led to a better MEMOTE score [103]. The only criterion for which *i*CN1361 scores weaker than RehMBEL1391 is the number of reactions, but this can be explained by the inclusion of many unconfirmed transport reactions with no associated genes in RehMBEL1391, while *i*CN1361 only included experimentally-supported transport reactions. In addition, *i*CN1361 was validated by comparing flux balance analysis (FBA) results to experimentally obtained results: the model predicted the growth phenotypes for different feedstocks with 87% accuracy, it accurately predicted the fluxes in the central carbon metabolism during growth on fructose, and it predicted gene essentiality with a performance of 92% [103]. *i*CN1361 therefore serves as a valuable resource for designing strategies to engineer *C. necator*.

##### Flux balance analysis

FBA is a constraint-based modelling method to determine the flux of metabolites in a metabolic network, the GSM, such that the biomass accumulation and target production rates can be predicted [104]. Using a modified version of the RehMBEL1391 GSM, FBA was used to identify rate-limiting reactions and thus propose candidate genes for overexpression to overcome these limitations [105]. Combined with elementary mode analysis, used to identify gene deletion candidates, FBA was used to design *C. necator* strains with a predicted autotrophic yield of 0.21–0.42 g/g for isobutanol and 0.20–0.34 g/g for hexadecanol [105]. FBA was also used to validate the *i*CN1361 GSM. It was used to predict *C. necator* growth on 131 different carbon sources, fluxes through central carbon metabolism for growth on fructose, and essentiality of genes for heterotrophic growth of *C. necator* [103]. Although the accuracy of FBA was high for these tests, the method is limited because it only accounts for the stoichiometry of metabolism. Thus, it leaves out many other factors that drive cellular processes.

##### Resource balance analysis

Resource balance analysis (RBA) is a constraint-based modelling method that adds the mass conservation of cellular resources as an additional constraint to FBA [106, 107]. RBA was applied to estimate and understand protein utilization in *C. necator* using the updated version of the RehMBEL1391 GSM. The authors input growth conditions with different substrates into the model and identified the proteins—enzymes and machinery—that were used by the cell under the given conditions [40]. The benefits of using RBA for this experiment were the increased accuracy of the model and the ability to predict the utilization of cellular machinery rather than just the metabolic reactions. However, many proteins were not modelled in the study, and although most of them were predicted to be unutilised, the gap suggests improvement of *C. necator* models is necessary. In addition, the study revealed that many proteins are under-utilised, which makes the protein seem inefficient [40]. This may also indicate that the current models are not well-suited to accurately representing *C. necator*.

## Conclusions

A goal of synthetic biology is the standardization and modularization of biological parts because it allows biology to be engineered in a straightforward manner that is transferable to a multitude of applications. The development of computational and experimental synthetic biology tools specific to *C. necator* has facilitated much more extensive engineering than was previously possible, and this has made its use as a biomanufacturing chassis much more feasible. However, *C. necator* is a relatively unique organism. As such, synthetic biology tools and methods should be *C. necator*-specific, instead of based on tools from other organisms. For example, effective and modular plasmids have been developed, but they rely on BHR origins of replication [56, 82, 85, 87–90]. Given that certain features of these biological parts have been shown to impede transformation efficiency [82], it would be advantageous to further our understanding of native origins of replication to utilize them in plasmid designs.

Pathway engineering could also benefit from the extensive physiological characterization done to date. One of the most discussed capabilities of *C. necator* is its ability to fix carbon as PHB. As such, the pathway for PHB production and degradation is well-studied and can inform metabolic engineering strategies, such as knocking out genes to reroute flux away from PHB production and towards a product of interest [72–75, 77]. Annotating and characterising the sequenced genome of *C. necator* is also important for developing effective pathway engineering strategies. Since the genes for converting citramalate to α-ketobutyrate were previously identified in *C. necator*, Wang et al. were able to design a simplified pathway towards production of isoleucine that required heterologous expression of only a single gene. The authors also utilised their knowledge of the cofactors necessary in the PHB synthesis pathway: knocking out the PHB pathway genes resulted in a cofactor imbalance that they rectified by choosing enzymes with a cofactor preference [75]. The success of these examples of using well-understood parts of *C. necator* physiology to inform engineering strategies serves as motivation for further studying and understanding lesser-known parts of its physiology that may prove useful in metabolic engineering.

The metabolic versatility of *C. necator* is one of the traits that makes it so attractive for biomanufacturing, but it also makes it difficult to use traditional engineering methods. The large fraction of under-utilised proteins and non-essential genes are suggested to be related to the environmental preparedness of *C. necator* [40]. In other words, the cell keeps a large reserve of proteins that are not necessary at a given point in time but may become useful if the conditions change. As such, the proteome appears to be inefficient and therefore to be a significant constraint on growth rate.

While it may seem like *C. necator* uses resources suboptimally, this is likely explained by the fact that it occupies a generalist niche, which requires robustness to changing environmental conditions. Therefore, our typical understanding of microbial physiology, based on fast-growing specialists, like *Escherichia coli*, may limit the applicability of existing models to *C. necator*. In a controlled culture, allocating resources to prepare for a changing environment hinders growth rate. As such, it is necessary to understand the control mechanisms that manage the metabolic flexibility of *C. necator* in order to tune and edit them to the needs of biomanufacturing rather than simply using *C. necator* as if it fits the mold of other chassis. Further exploration of the physiology of *C. necator* under varying feeding conditions could help identify these mechanisms of interest.

In addition, models that go beyond constraints to include kinetics and thermodynamics could also help identify the optimisation that takes place in *C. necator*. To switch between substrates based on availability, the thermodynamic landscape of metabolic reactions in *C. necator* must change according to metabolite concentrations [108, 109]. Therefore, a model considering elementary flux modes and max–min driving force analysis can help identify limitations in metabolic engineering strategies, as was done by Janasch et al. when they identified low driving forces through citrate lyase and aconitase and cofactor balancing as limitations of formate utilization for PHB production [109]. Thus, to achieve more effective modifications of *C. necator*, continued development of experimental and computational tools is required.

We must also be careful not to overengineer *C. necator* to fit applications that do not fall within its niche. Autotrophic growth on CO_2_ differentiates *C. necator* from other chassis, so this ability should be a focus of engineering strategies going forward. Instead of developing heterotrophic metabolism for a given application before switching to autotrophic metabolism, the design strategy should involve and optimise CO_2_ consumption from the start. This optimisation may involve using a mixotrophic approach that would provide additional reducing power beyond H_2_.

Further strain optimisation efforts should focus on engineering *C. necator* away from a generalist metabolism toward high production in autotrophic conditions to support the movement toward industrial application of this organism. This change will likely require genome reduction to reduce protein burden resulting from genomic redundancy and from environmental readiness. Additionally, decoupling production from nutrient limitation may be necessary to develop an industrial *C. necator* strain because production paired with balanced growth is expected to simplify the fermentation process and improve productivity. The *C. necator* stress response is a potential target for alleviating the nutrient-limitation requirement for production. Continued exploratory research into *C. necator* would also benefit these engineering strategies, as a physiology-informed approach should yield the most effective methods by making the best use of the features that naturally make *C. necator* such an interesting chassis.

## Data Availability

No datasets were generated or analysed during the current study.
